# Cost-effectiveness of approaches to cervical cancer screening in Malawi: comparison of frequencies, lesion treatment techniques, and risk-stratified approaches

**DOI:** 10.1186/s12913-024-11226-2

**Published:** 2024-07-09

**Authors:** Petra W. Rasmussen, Risa M. Hoffman, Sam Phiri, Amos Makwaya, Gerald F. Kominski, Roshan Bastani, Agnes Moses, Corrina Moucheraud

**Affiliations:** 1 RAND Corporation, 1776 Main Street, Santa Monica, CA 90401 USA; 2 David Geffen School of Medicine, Division of Infectious Disease, University of California Los Angeles, 885 Tiverton Drive, Los Angeles, CA 90095 USA; 3 Partners in Hope, Area 36 Plot 8, Lilongwe, Malawi; 4 Fielding School of Public Health, Department of Health Policy and Management, University of California Los Angeles, 650 Charles E Young Dr S, Los Angeles, CA 90095 USA; 5 School of Global Public Health, Department of Public Health Policy and Management, New York University, 708 Broadway, New York, NY 10003 USA

**Keywords:** Cervical cancer, Global health, Cost-effectiveness, Health policy

## Abstract

**Background:**

Recently-updated global guidelines for cervical cancer screening incorporated new technologies—most significantly, the inclusion of HPV DNA detection as a primary screening test—but leave many implementation decisions at countries’ discretion. We sought to develop recommendations for Malawi as a test case since it has the second-highest cervical cancer burden globally and high HIV prevalence. We incorporated updated epidemiologic data, the full range of ablation methods recommended, and a more nuanced representation of how HIV status intersects with cervical cancer risk and exposure to screening to model outcomes of different approaches to screening.

**Methods:**

Using a Markov model, we estimate the relative health outcomes and costs of different approaches to cervical cancer screening among Malawian women. The model was parameterized using published data, and focused on comparing “triage” approaches—i.e., lesion treatment (cryotherapy or thermocoagulation) at differing frequencies and varying by HIV status. Health outcomes were quality-adjusted life years (QALYs) and deaths averted. The model was built using TreeAge Pro software.

**Results:**

Thermocoagulation was more cost-effective than cryotherapy at all screening frequencies. Screening women once per decade would avert substantially more deaths than screening only once per lifetime, at relatively little additional cost. Moreover, at this frequency, it would be advisable to ensure that all women who screen positive receive treatment (rather than investing in further increases in screening frequency): for a similar gain in QALYs, it would cost more than four times as much to implement once-per-5 years screening with only 50% of women treated versus once-per-decade screening with 100% of women treated. Stratified screening schedules by HIV status was found to be an optimal approach.

**Conclusions:**

These results add new evidence about cost-effective approaches to cervical cancer screening in low-income countries. At relatively infrequent screening intervals, if resources are limited, it would be more cost-effective to invest in scaling up thermocoagulation for treatment before increasing the recommended screening frequency. In Malawi or countries in a similar stage of the HIV epidemic, a stratified approach that prioritizes more frequent screening for women living with HIV may be more cost-effective than population-wide recommendations that are HIV status neutral.

## Background

Cervical cancer causes substantial mortality and morbidity worldwide [1], especially in low- and middle-income countries (LMICs), where approximately 90% of the 603,000 new cervical cancer cases and 341,000 attributable deaths occur [2]. In Africa, cervical cancer is the leading cause of cancer death for women [3]. Although a significant portion of cervical cancer mortality can be effectively averted through routine screening [4–6], screening programs face implementation challenges in constrained health systems due to lack of trained health workers, laboratory infrastructure and personnel, and equipment and supplies to conduct these procedures [7, 8]. For these reasons, many women in low-income countries do not receive routine screening [9] and therefore present with late-stage cancer that is not treatable [10]. As a result, the burden of cervical cancer continues to fall disproportionately on women in LMICs [11–13].

Countries with generalized HIV epidemics may need specialized screening strategies for women living with HIV, as there are important synergies between HIV and cervical cancer [14, 15]. The virus that causes the majority of cervical cancer cases (human papillomavirus, HPV) is also sexually transmitted [16], so the diseases share common risk factors. Women living with HIV are more likely to be infected with HPV, to have persistent HPV infection, and to be infected with multiple and higher-risk HPV types [17, 18]. However, as the HIV epidemic evolves, the relationship between HIV and cervical cancer is changing as well. A recent meta-analysis found that initiation and adherence to antiretroviral therapy (ART) significantly reduces the incidence and progression of cervical lesions and neoplasia, and ultimately of cervical cancer [19]. Therefore, incorporating HIV clinical characteristics and treatment history into representations of women’s HIV status may more effectively define risk-stratifying screening recommendations, rather than looking at HIV status as a homogeneous risk factor.

The World Health Organization (WHO) recently changed its cervical cancer screening recommendations, from a single-visit “screen and treat” strategy in resource-limited settings [20], to using HPV DNA detection as the primary approach to cervical cancer screening, in either a “screen and treat” approach (with treatment immediately following a positive HPV DNA result) or a “screen, triage, and treat” approach (with an additional screening test following a positive HPV DNA result, and treatment accordingly) [21]. These recommendations let countries determine for themselves which triage test is used (for example, whether cytology or visual inspection with acetic acid [VIA]), and which ablative treatment is used (cryotherapy or thermal coagulation). In countries where HPV DNA testing is not widely available, the WHO guidelines continue to recommend primary screening using either cytology or visual inspection with acetic acid (VIA). As many LMICs’ health systems lack the requisite infrastructure to support HPV DNA testing at-scale [22], VIA is likely to persist as a mainstay to primary screening until infrastructure, financing, human resource and other systems-level constraints to HPV DNA testing at-scale can be resolved [23]. There are currently very few cervical cancer prevention policies/guidelines in Africa that include HPV DNA testing [24].

Malawi has the second-highest age-standardized mortality rate attributable to cervical cancer in the world [3, 12]. Malawi also has substantial HIV burden, with a prevalence of 11.0% among adult women (of whom approximately 87% are on ART) [25], and approximately 40% of adult women living with HIV in Malawi may be infected with at least one high-risk HPV type [26, 27]. According to surveys, fewer than 10% of women in Malawi report ever being screened for cervical cancer [9, 26, 28]. Treatment of precancerous lesions among women completing screening is also suboptimal: in 2015, only 41% of Malawian women who had lesions eligible for removal received this service [29]. Malawi’s current National Cervical Cancer Strategy aims to screen at least 80% of women aged 25–49 years who have never been screened before, and treat 90% of women who screen positive [29]. The cervical cancer program guidelines from the Malawi Ministry of Health recommend beginning routine screening at age 25, with repeat screens every year for women living with HIV and every 3 years for women without HIV until age 50 [30].

This study assesses the potential impact and cost-effectiveness of approaches to the secondary prevention of cervical cancer in Malawi. The main comparisons are between lesion treatment techniques (cryotherapy versus thermocoagulation) and between screening schedules (annual, biannual, triannual, etc.); and we stratify these by HIV status. These comparisons were selected because they are implementation options left to countries’ discretion in the latest WHO recommendations. This model does not include HPV DNA testing in its screening cascade because many LMICs are not yet able to implement this for primary screening (as described above).

We first modeled the potential relative health impacts (quality-adjusted life years gained, and deaths averted) and cost-effectiveness (dollars per quality-adjusted life year) of implementing different cervical cancer screening strategies in Malawi. Then, we estimated the budget impact of implementing these strategies in Malawi. Such modeling analyses have previously provided critical inputs into cervical cancer prevention policies (e.g., screening frequencies) [31], and we hope these results can similarly help inform policies in high-burden African settings where modeling has been relatively less influential on policy and clinical guidelines.

## Methods

### Description of the model

We constructed a Markov model to estimate the health outcomes and costs of different scenarios for cervical cancer screening and treatment among adult women in Malawi. The model began with women at age 30, to reflect World Health Organization guidelines [32]. A Markov model was selected because it is data-efficient (i.e., can use a relatively small set of input parameters which can be obtained from secondary or published data, without needing a high degree of disaggregation) and because its decision tree structure can be readily presented, replicated, and explained to diverse audiences without specific technical modeling expertise such as policymakers, clinicians, and public health researchers.

**Fig. 1:**
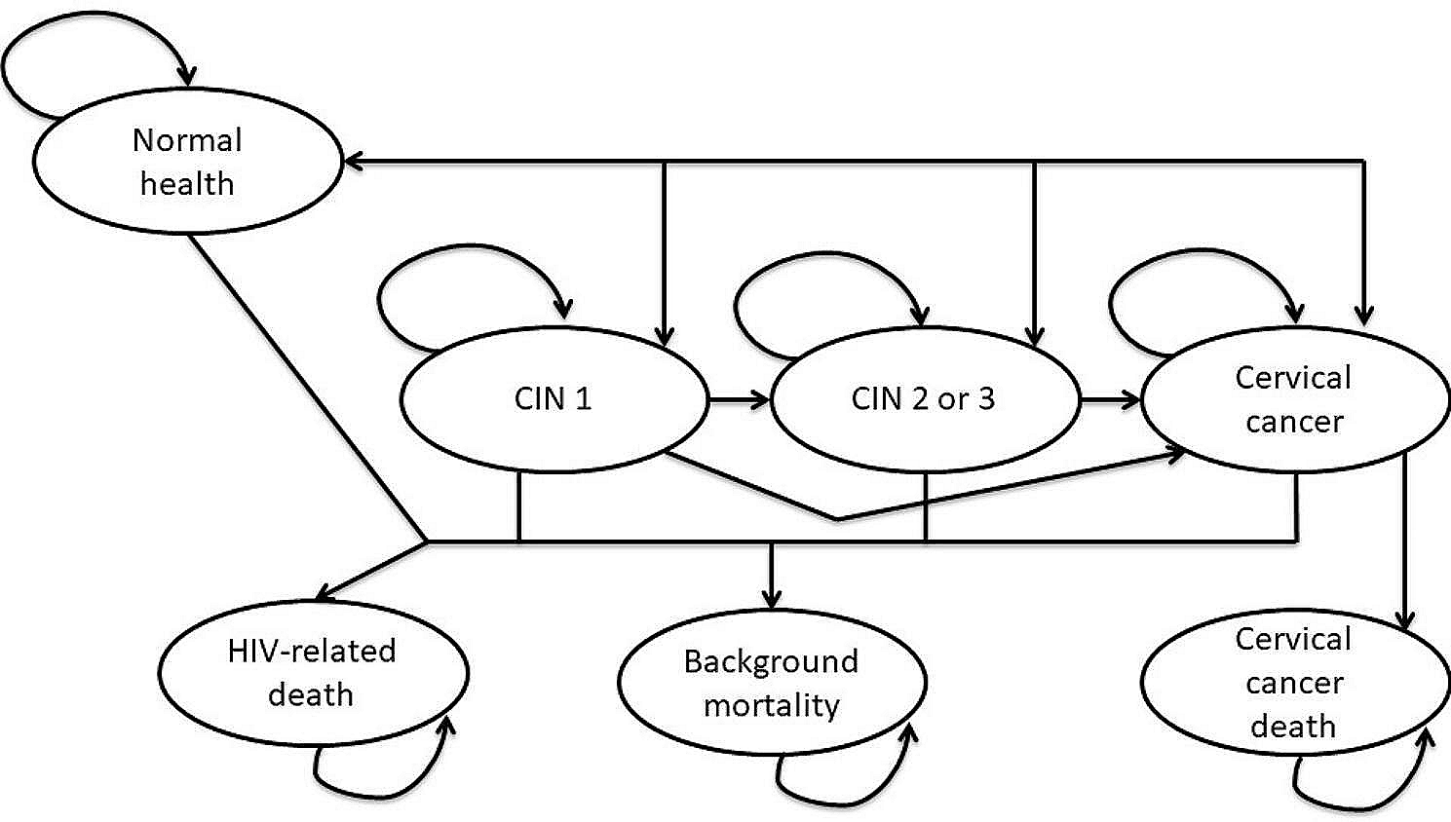
Possible transitions between health states included in Markov model

Health states in the model included: (1) normal health; (2) cervical intraepithelial neoplasia grade 1 (CIN 1); (3) CIN 2 or CIN 3; (4) living with cervical cancer; and (5) mortality. Mortality could be associated with cervical cancer, with HIV, or due to any other cause. Figure 1 illustrates the monthly transition possibilities in the model. Women could progress from normal health to CIN 1, CIN 2 or 3, cervical cancer, or could die from HIV-related or other non-cervical cancer-related causes. The model also allows for return to healthier states. Depending on treatment status, those with CIN 1 could regress to normal health or progress to CIN 2 or 3, cervical cancer, or die from HIV-related or other non-cervical cancer related causes. Similarly, those with CIN 2 or 3 could regress to CIN 1 or normal health (with treatment) or could progress to cervical cancer or could die from HIV-related or other non-cervical cancer causes. Only individuals with cervical cancer could progress to cervical cancer-related death. Individuals with cervical cancer who receive cancer treatment could regress to normal health. In addition, those with cervical cancer regardless of treatment status could die from HIV-related (if living with HIV) or other causes.

Each month, for the first 20 years of the simulation, women in the normal, CIN 1, and CIN 2 or 3 arms were eligible to be screened for cervical cancer, or they could go unscreened. The main analyses use screening with VIA. Those who screened positive (regardless of whether it was a true or false positive) could then receive no treatment, or treatment in the form of either cryotherapy or thermocoagulation. Women who developed cervical cancer could receive no treatment or could receive curative treatment or palliative care. For the last 10 years of the simulation, once the cohort had aged beyond the recommended guidelines for screening, no screenings were performed but health benefits from previous screenings continued to accrue.

Costs and benefits were discounted at an annual rate of 3% (0.254% monthly), and half-cycle corrections to account for the possibility that transitions between states can occur at any time during the month. We ran the model for 360 cycles, or 30 years. Health outcomes were measured in QALYs and were based on values used in previous studies [33, 34]. Costs were reported in 2020 US dollars. Incremental cost-effectiveness ratios (ICERs) were calculated as the difference in health outcomes and costs between strategies. All models were created and analyzed using TreeAge Pro 2021.

### Model calibration

The baseline model was calibrated based on the expected number of cervical cancer cases among 30- to 39-year old women over a 30-year period, using data from the Malawi Cancer Registry [35] and estimates from GLOBOCAN [36] creating a calibration target between 60 and 200 cases per year. We compared our year 1 baseline results after running the model and found that they were near this range (252 cervical cancer cases), giving us confidence that the model was well-calibrated.

**Table 1:** Model parameters

	Women without HIV	Women living with HIV			Source
		CD4 > 500	CD4 200–500	CD4 < 200	
Starting age	30	30	30	30	
Number of women in Malawi, age 30	78,668	9,466	7,381	2,012	[28]
Number of women in Malawi, age 30, on ART	0	7,951	6,200	1,690	
Number of women in Malawi, age 30, not on ART	0	1,514	1,181	322	
**Beginning health states**					
Normal	0.963	0.963	0.883	0.793	[34]
CIN 1	0.024	0.024	0.096	0.165	[34]
CIN 2/3	0.013	0.013	0.02	0.04	[34]
Cervical cancer	0	0	0.001	0.002	[34]
Cervical cancer mortality	0	0	0	0	n/a
HIV-related mortality	0	0	0	0	n/a
Background mortality	0	0	0	0	n/a
**Care coverage (for women not on ART; increased by 50% for those on ART)**					
VIA	0.002	0.002	0.002	0.002	[37]
Thermocoagulation or cryotherapy	0.421	0.421	0.421	0.421	[37]
Curative cancer treatment	0.01	0.01	0.01	0.01	Author estimate
Palliative cancer treatment	0.05	0.05	0.05	0.05	Author estimate
**Test sensitivity and specificity**					
VIA, true negative^a^	0.75	0.75	0.75	0.75	[38, 39]
VIA, false negative^b^	0.4	0.4	0.4	0.4	[38, 39]
**Mortality transitions**					
HIV-related mortality^c^	0	0	0.001	0.003	[40]
Background mortality	0.001	0.001	0.001	0.001	[41]
**Normal arm health state transitions**					
Progress to CIN 1	0.0002	0.0002	0.007	0.007	[34]
Progress to CIN 2/3	0.000001	0.000001	0.0002	0.0002	[34]
Progress to cervical cancer	0.00000004	0.00000004	0.000006	0.000006	[34]
**CIN 1 arm (without treatment) health state transitions**					
Regress to normal	0.0299	0.0299	0.0299	0.0299	[38]
Progress to CIN 2/3	0.0073	0.0073	0.0293	0.0293	[34]
Progress to cervical cancer	0.0002	0.0002	0.0009	0.0009	[34]
**CIN 1 arm (with treatment) health state transitions**					
Regress to normal	0.94	0.94	0.94	0.94	[42]
Progress to CIN 2/3	0.0004	0.0004	0.0018	0.0018	[34]
Progress to cervical cancer	0.00001	0.00001	0.00006	0.00006	[34]
**CIN 2/3 arm (without treatment) health state transitions**					
Regress to normal	0.003	0.003	0.003	0.003	[34]
Regress to CIN 1	0	0	0	0	[34]
Progress to cervical cancer	0.02	0.02	0.0242	0.0242	[34]
**CIN 2/3 arm (with treatment) health state transitions**					
Regress to normal	0.94	0.94	0.94	0.94	[42]
Regress to CIN 1	0	0	0	0	[34]
Progress to cervical cancer	0.0012	0.0012	0.0015	0.0015	[34]
**Cervical cancer (palliative or no treatment) health state transitions**					
Regress to normal	0	0	0	0	[38]
Regress to CIN 1	0	0	0	0	[38]
Regress to CIN 2/3	0	0	0	0	[38]
Cervical cancer death^d^	0.0923	0.0923	0.0923	0.0923	[38]
**Cervical cancer (curative treatment) health state transitions**					
Regress to normal	0.15	0.15	0.15	0.15	[38]
Regress to CIN 1	0	0	0	0	[38]
Regress to CIN 2/3	0	0	0	0	[38]
Cervical cancer death	0.0093	0.0093	0.0093	0.0093	[38]
**Costs**					
Thermocoagulation	$3.66				[43]
VIA	$1.25				[44]
Cryotherapy	$9.52				[43]
Cytology *(sensitivity analysis)*	$13.64				[44]
Curative cervical cancer treatment^e^	$1,054				[45]
Palliative care	$1.00				Author estimate
**QALYs**					
Normal	1				
False positive for CIN 1, 2/3	0.97				[33]
Treatment for CIN 1	0.97				[33]
Treatment for CIN 2/3	0.93				[33]
Cervical cancer	0.5				[34]
Death	0				n/a

Note: Transition probabilities represent monthly probabilities

^a^ True negative rate is equivalent to the test specificity

^b^ False negative rate is equivalent to 1 minus the test sensitivity

^c^ HIV-related mortality calculated based on the number of AIDS deaths in Malawi by CD4 count, age, and sex

^d^ Based on 69% mortality rate

^e^ Curative cancer treatment includes costs associated with both surgery and radiotherapy [46]

### Model inputs: clinical and epidemiological

Table 1 details all model probabilities (initial and transitional), and costs. Reflecting statistics on HIV prevalence and treatment in Malawi [28], 80.7% of the cohort of 97,527 women age 30 years old was assumed to be women without HIV and 19.3% women living with HIV; among those with HIV, 10.7% had a CD4 count below 200 cells/mm^3^, 39.1% had a CD4 count between 200 and 500 cells/mm^3^, and 50.2% had a CD4 count higher than 500 cells/mm^3^. Within each CD4 count group, women were then categorized as either being on ART or not per data from the household-level, nationally-representative Malawi Population-based HIV Impact Assessment (MPHIA) survey [28]. After the initial distribution into groups by HIV status, CD4 count, and ART use, women did not move between these cohorts over the period of analysis. The probability of dying from an HIV-related cause differed by CD4 count: 0% of HIV-negative women and women with a CD4 count higher than 500 cells/mm^3^, 1% of women with a CD4 count between 200 and 500 cells/mm^3^, and 3.8% of women with a CD4 count less than 200 cells/mm^3^ dying annually [40]. Similarly, disease progression differed by CD4 count. Women with a CD4 count of 500 or lower had higher probabilities of cervical disease progression than women without HIV and women with a CD4 count higher than 500 cells/mm^3^ [34].

### Model inputs: costs

We calculated the per procedure costs for thermocoagulation and cryotherapy. The cryotherapy and thermocoagulation costs were based on the treatment experience in Kenya (due to the lack of available information from Malawi) and include personnel time and supplies. For cryotherapy, we calculated an overall total for the cost of the cryotherapy machine, tips, nitrous oxide or carbon dioxide tank, and gas refills. For thermocoagulation, we calculated an overall total for the cost of the thermocoagulation machine, including 2 coagulator probes, and an additional battery. These overall costs were converted into per procedure costs with the assumption that the machines would last for 5 years being used 5 days a week with 6 treatments per day (author assumption based on information shared by a cervical cancer program in Malawi). Cryotherapy treatment for lesions cost $9.52 per procedure and thermocoagulation cost $3.66 per procedure. We also included costs for VIA ($1.25), curative cervical cancer treatment ($1,054), and palliative cervical cancer treatment ($1.00) based on the literature [44, 45].

### Model inputs: baseline care coverage

Under the baseline scenarios, eligible women who were not on ART had a 0.2% monthly probability of being screened [29] and women on ART had a 0.3% monthly probability of being screened (the difference in screening coverage between groups was based on author estimate). Women who screened positive had a 42.1% probability of receiving thermocoagulation or cryotherapy [29]. We assumed that each month, 5% of women who developed cervical cancer received palliative treatment, 1% received curative treatment, and 94% received no treatment.

### Scenarios for analysis

We ran several scenarios to test policies aimed at increasing cervical cancer screening and precancerous lesion treatment. First, we increased VIA screening from the baseline up to an annual screen for all women: once per lifetime, once every 10 years, once every 3 years, once every 2 years, and once per year. Second, we investigated the impact of lesion treatment technologies; and of scaling up treatment for all women who screen positive for lesions, from 50 to 75 to 100% (the latter is termed “universal treatment” herein, i.e., all women who require treatment receive it). Third, we adjusted screening frequency for women with versus without HIV at different rates, as Malawian clinical guidelines recommend more frequent screenings for women with HIV.

### Sensitivity analyses

Given uncertainty in some parameter estimates, we assessed the extent to which our main findings were influenced by precision in these values. We examined the sensitivity of our results to differing values for cost, test sensitivity and specificity, and treatment effectiveness.

## Results

### Comparing methods for screening and lesion treatment

At all proposed screening frequencies for women in Malawi, cryotherapy was more expensive and no more effective than thermocoagulation (Table 2). Increased frequency of screening substantially increased estimated costs per QALY with both thermocoagulation or cryotherapy – and the added cost of cryotherapy became particularly high at more-frequent screening frequencies. Annual screening is particularly expensive, at $7.33/QALY if thermocoagulation is used, or $12.41/QALY if cryotherapy is used. The gained QALYs for these scenarios (16.77) represent only a very small health benefit over biannual screening (16.74 QALYs), but substantially more cost.

**Table 2:** Costs and outcomes for different screening frequencies and lesion treatment options (using a “screen and treat” strategy with VIA, assuming universal uptake of treatment)

	Thermocoagulation	Cryotherapy
Current care (base case)	$0.34/ QALY *(= $5.53/16.29 QALYs)*	$0.37/ QALY *(= $6.09/16.29 QALYs)*
Screen once per lifetime	$0.53/ QALY *(= $8.64/16.40 QALYs)*	$0.73/ QALY *(= $12.01/16.40 QALYs)*
Screen every 10 years	$1.02/ QALY *(= $16.94/16.54 QALYs)*	$1.63/ QALY *(= $26.96/16.54 QALYs)*
Screen every 5 years	$1.80/ QALY *(= $30.02/16.64 QALYs)*	$2.99/ QALY *(= $49.72/16.64 QALYs)*
Screen every 3 years	$2.83/ QALY *(= $47.28/16.70 QALYs)*	$4.75/ QALY *(= $79.33/16.70 QALYs)*
Screen every 2 years	$4.06/ QALY *(= $67.94/16.74 QALYs)*	$6.84/ QALY *(= $114.55/16.74 QALYs)*
Screen annually	$7.33/ QALY *(= $123.00/16.77 QALYs)*	$12.41/QALY *(= $208.04/16.77 QALYs)*

**Fig. 2:**
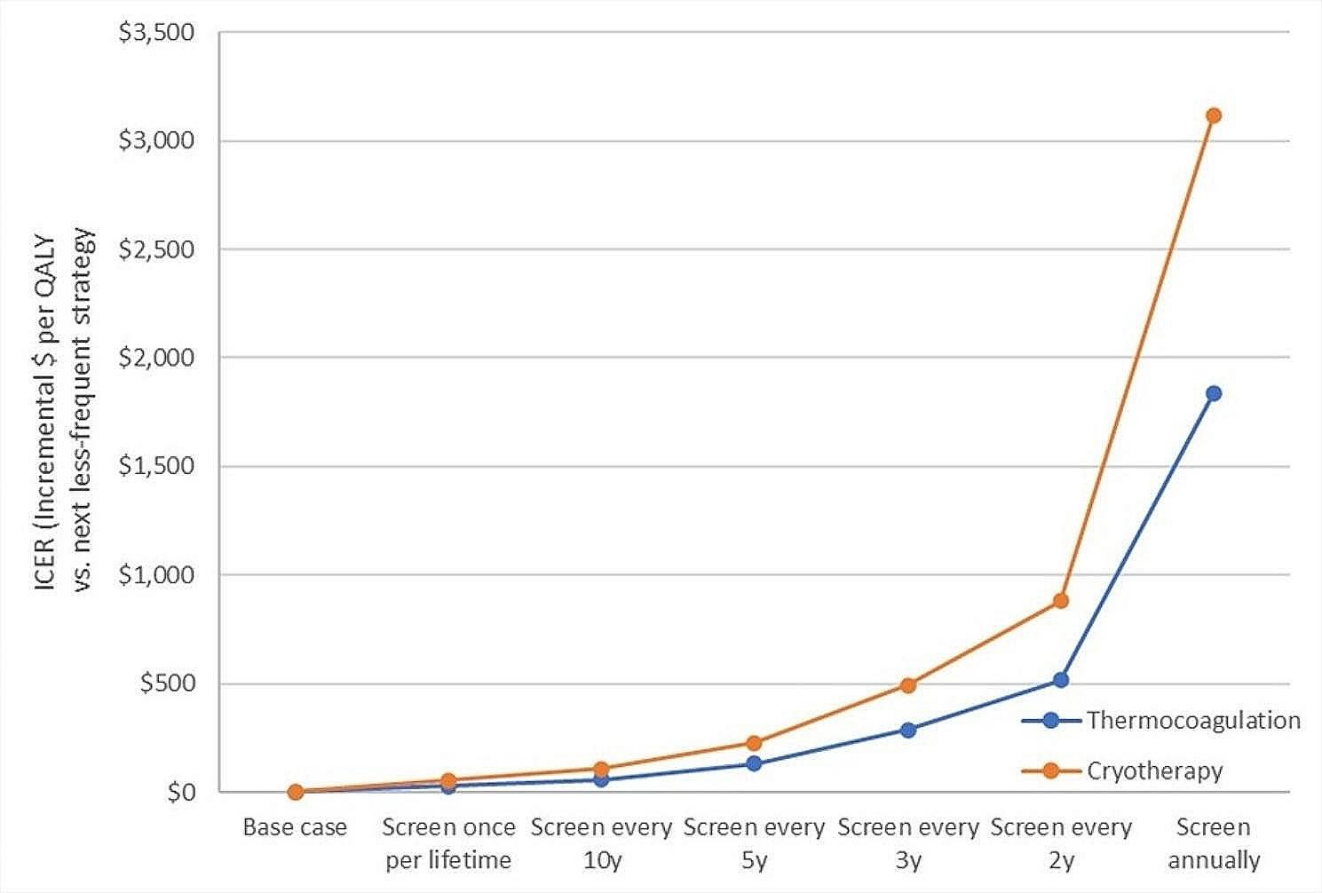
Incremental cost-effectiveness ratios (ICERs): costs per QALY gained comparing screening frequencies and lesion treatment approaches Note: Sample includes all women in the model

Examining the incremental costs and benefits of moving to more-frequent screening intervals (i.e., the incremental cost-effectiveness ratio [ICER] of a more-frequent schedule compared to a less-frequent schedule), there are two inflection points (Fig. 2). Using either thermocoagulation or cryotherapy for lesion treatment, moving to once-lifetime or once-per-decade frequency would add very little cost per QALY gained: once-lifetime screening would cost an additional $28 per QALY gained if thermocoagulation is used, or $54 if cryotherapy is used; and, compared to this, once-per-decade would cost an additional $59 for thermocoagulation, or $107 for cryotherapy, per QALY gained. There is a slight increase if screening were instead offered every 5 or 3 years – and, beyond this, incremental costs per QALY rise quickly. If screening were offered annually rather than biannually, this would cost an additional $1835 for each QALY gained if thermocoagulation is used, or an additional $3116 if cryotherapy is used.

### Comparing screening frequency and treatment coverage

**Fig. 3:**
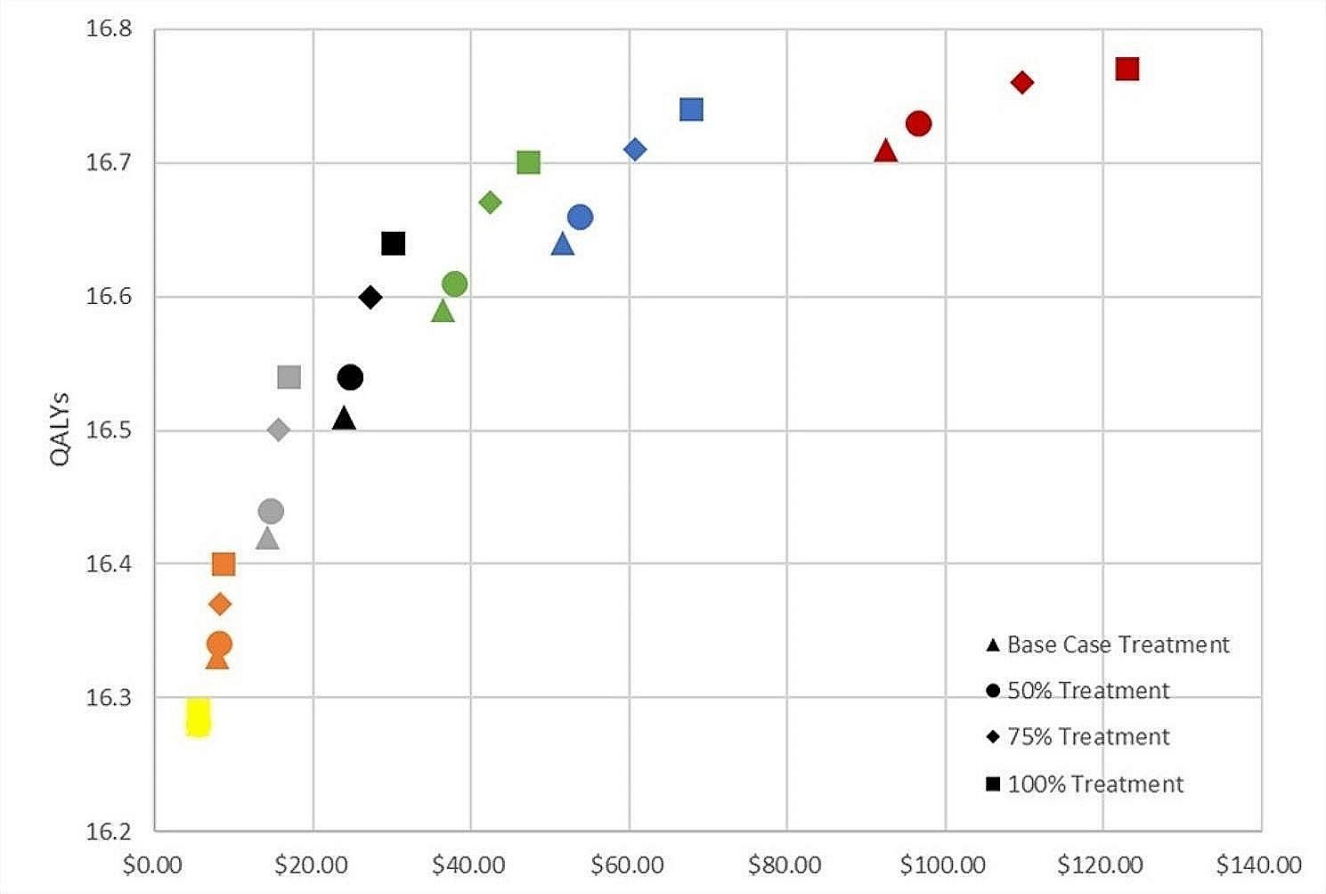
Efficiency frontier for screening frequency and treatment uptake (using a “screen and treat” strategy with VIA and thermocoagulation) Note: Yellow = base case screening; Orange = screened once in lifetime; Grey = screened every 10 years; Black = screened every 5 years; Green = screened every 3 years; Blue = screened every 2 years; Maroon = screened every year

We assessed the relative benefits and costs of investing in more frequent screening versus better coverage of lesion treatment among those women eligible (Fig. 3). Particularly at low screening frequencies (once per lifetime, once per decade), investing in better treatment coverage results in health benefit gains with relatively low additional cost. Screening once every 10 years but only treating 50% of eligible women would cost $14.59 for 16.44 QALYs – and increasing this treatment coverage to 100% at this same screening frequency would cost $16.94 for 16.54 QALYs. From here, increasing screening frequency to once every 3 years and maintaining the lower treatment coverage (50%) would cost $37.85 for 16.61 QALYs, and universal treatment at this screening frequency would cost $47.28 for 16.70 QALYs. The “flatter” arcs for treatment scale-up at annual and biannual screening frequencies (colors blue and red) indicate the relatively lower health gains per dollar spent.

Investing in universal treatment would avert approximately twice as many deaths at low screening frequencies versus baseline treatment coverage (Table 3). This marginal benefit decreases as screening frequencies increase, although ensuring universal treatment always saves more lives than using baseline treatment coverage at every screening frequency.

**Table 3:** Cervical cancer deaths averted in this cohort (compared to base case) after 30 years, using VIA and thermocoagulation

	Baseline treatment coverage: Cervical cancer deaths averted over 30-year period	Universal treatment: Cervical cancer deaths averted over 30-year period
Screen once per lifetime	433 deaths averted (7.6%)	1045 deaths averted (18.8%)
Screen every 10 years	1410 deaths averted (24.9%)	2391 deaths averted (43.0%)
Screen every 5 years	2234 deaths averted (39.4%)	3330 deaths averted (59.9%)
Screen every 3 years	2942 deaths averted (52.0%)	3844 deaths averted (69.2%)
Screen every 2 years	3434 deaths averted (60.6%)	4119 deaths averted (74.1%)
Screen annually	4014 deaths averted (70.9%)	4387 deaths averted (78.9%)

Note: The number of cervical cancer deaths under the baseline screening with baseline treatment was 5,663 over a 30-year period. The number of cervical cancer deaths under baseline screening with universal treatment was 5,558 over a 30-year period

### Risk-stratified screening approaches

**Fig. 4:**
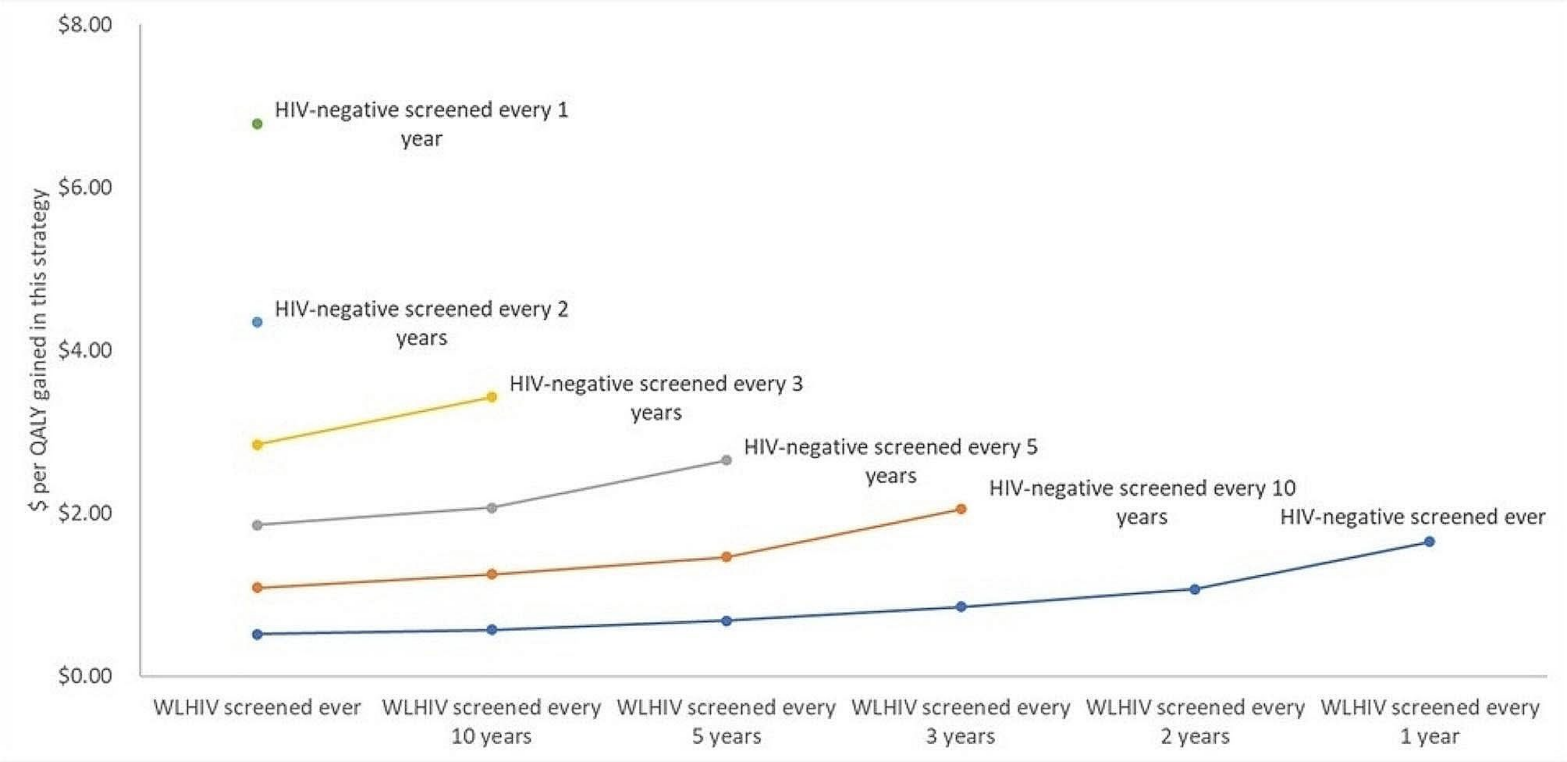
Risk-stratified screening frequency by HIV status (using a “screen and treat” strategy with VIA and thermocoagulation, assuming universal uptake of treatment) Note: WLHIV = women living with HIV

We tested the potential population-level costs and impacts of stratifying screening strategies by women’s HIV status – namely increasing frequency for women living with HIV ﻿– using “screen and treat” with VIA and thermocoagulation (Fig. 4). Differential screening strategies offer more health gains per dollar spent: if women living with HIV in Malawi were screened annually while women without HIV were screened biannually, this would have similar health effects as annual screening for all (16.75 to 16.77 QALYs) but would only cost $72.75 per woman to attain this, versus $113.78 per woman if everyone were screened annually. The current recommendation in Malawi, to screen women with HIV every year and women without HIV every 3 years, would cost $57.29 per 16.73 QALYs gained.

### Budget impact analysis

This model estimates that the status quo level of “screen and treat” in Malawi costs approximately $530,860 for the cohort of 97,527 30-year old women over 30 years, or $5.44 per woman. The total cost of universal once-per-lifetime screening (including ensuring that each woman who screens positive receives thermocoagulation treatment) would be approximately $826,186, or an additional $295,326 over baseline across the 30-year period. If instead these women were screened once per 3-year period and all screen-positive women receive thermocoagulation treatment, this would cost an additional $3,756,061 over the 30-year period (compared to the status quo). The “screen and treat” frequency recommended in current Malawi clinical guidelines – every year for women living with HIV and every 3 years for women without HIV – would cost $4,235,723 under baseline treatment assumptions, or $5,589,919 with universal treatment.

### Sensitivity analysis results

We assessed the sensitivity of our main results to the input parameter values using one-way sensitivity analyses. In these analyses we included the cost of VIA, the cost of treatment (either thermocoagulation or cryotherapy), the probability of receiving a false negative from VIA screen, the probability of receiving a true negative from VIA screen (specificity), and the probability of treatment curing a lesion. The results were most influenced by the cost of screening (Table 4). The second-most influential parameter was the specificity of screening. We also evaluated frequencies and lesion treatment techniques using a two-visit strategy with cytology-based testing (data not shown); this was more expensive and not more effective at any screening frequency.

**Table 4:** Sensitivity analysis of key variables included in model

Thermocoagulation (for reference, average cost under the base case scenario: 5.46)			
*Variable*	*Variable range*	*High*	*Low*
Cost of VIA	$1 - $5	6.7858	5.3697
VIA, true negative (specificity)	0.2–1	5.3257	5.7498
Cost of thermocoagulation	$1 - $5	5.3523	5.5116
VIA, false negative	0.24–1	5.4235	5.5351
Treatment curing lesion	0.9–0.99	5.4532	5.4622

Cryotherapy (for reference, average cost under the base case scenario: 5.69)			
*Variable*	*Variable range*	*High*	*Low*
Cost of VIA	$1 - $5	5.6031	7.0192
VIA, true negative (specificity)	0.2–1	5.3468	6.4501
Cost of thermocoagulation	$1 - $15	5.3523	5.9098
VIA, false negative	0.24–1	5.6628	5.7555
Treatment curing lesion	0.9–0.99	5.6866	5.6956

## Discussion

These findings add to a growing literature demonstrating that screening for the secondary prevention of cervical cancer is a highly cost-effective intervention [33]. “Screen and treat” is relatively inexpensive and has the potential to result in substantial health benefits. Similar to previous studies, we find that very frequent screening (e.g., annual or biannual) is not optimal for maximizing health benefits (regardless of women’s HIV status) and is very costly in resource-constrained contexts. This model was built to reflect the clinical and epidemiologic context of Malawi but the results may have relevance for other lower-resource settings with similar burdens of HIV and similar costs of implementing “screen and treat” programs.

This paper also adds new information to the evidence base. First, to our knowledge this is the first analysis that evaluates thermocoagulation, and compares it to cryotherapy. We find that thermocoagulation is a cost-effective approach. The difference in cost between thermocoagulation and cryotherapy is largely driven by the more expensive supplies associated with cryotherapy. The cryotherapy machine itself is more expensive and then requires cryotherapy tips, a gas tank, and gas refills. By comparison, the thermocoagulation machine comes with two probes and only requires an additional battery. If women were screened decennially, the two approaches would result in similar health gains but cryotherapy would cost 60% more per QALY than thermocoagulation (US$ 1.63/QALY and US$ 1.02/QALY, respectively). These cost savings become particularly noticeable at higher screening frequencies. We also evaluated the costs and impacts of cytology-based screening – but its high cost makes it a less favorable choice at any screening frequency. We do not compare the ICER results of this model to any willingness-to-pay thresholds given recent discussions about the relevance of such thresholds particularly in low-resource settings [51]. Rather, we hope these comparative analyses can inform policy and practice by indicating where greater (or lesser) investments or attention should be paid, and by estimating the overall budget impact of different approaches. These results may therefore offer informative insights for countries considering whether to incorporate thermocoagulation into their cervical cancer prevention programs.

Second, we undertook a novel analysis of investing in screening versus treatment. A successful cervical cancer prevention program should ideally increase both screening uptake and uptake of lesion treatment for women who are eligible; but, if resources are limited, policymakers may need to phase in their investments. Another recent analysis from Uganda found that policies that focus on increasing access to screening for previously-unscreened women may be more equitable and efficacious than a policy that focuses on increasing frequency for those women who are already getting screened [52]. We focus on a different tradeoff here and find that at relatively infrequent screenings (e.g., once per lifetime or once per decade) it is more impactful and less expensive to scale up thermocoagulation coverage rather than invest in increased screening frequency. If all women were screened only once per lifetime, ensuring universal treatment would avert twice as many deaths as would occur in status quo. We acknowledge that truly universal treatment (i.e., every woman with a positive screening result receives treatment) may be too ambitious, but the model also shows gains with each increase in treatment coverage (from 50 to 75 to 100%) so therefore recommend that expanding treatment should be a top policy and program priority for countries like Malawi where most women are screened on a relatively infrequent schedule. This will require investments into strengthening infrastructure (facilities able to offer same-day treatment for eligible women – i.e., have functional and available equipment), human resources skilled at delivering treatment services, and tracing and referral programs for women needing follow-up. As numerous studies have identified highly variable rates of follow-up after a positive cervical cancer screening result [53–55], these results may be relevant to numerous other settings where loss to follow-up is a substantial challenge.

Third, we quantified the benefits of risk-stratifying screening recommendations by HIV status: differential screening frequencies (more often for women living with HIV and less often for women without HIV) are more cost-effective than homogeneous screening frequencies for all women. Few prior cost-effectiveness analyses have considered the role of HIV when comparing cervical cancer control strategies even in high HIV-burden settings [56], even though women living with HIV are disproportionately affected by cervical cancer [57, 58]. Very few countries in Africa currently have cervical cancer policies with risk-stratified screening algorithms, so these results may lend support for expanding this approach. Countries that receive HIV prevention and treatment funds from the U.S. President’s Emergency Plan for AIDS Relief (PEPFAR) are also supported to integrate cervical cancer screening into HIV care and treatment programs [59], so are well-positioned to implement this. It is beyond the scope of this analysis to recommend a “right” screening approach for other settings – but these results do suggest that different screening frequencies based on HIV status may be a more cost-effective use of resources than a status-neutral screening schedule in high HIV burden, low-resource settings like Malawi.

Although this model lacks some of the analytic complexity of other recent modeling efforts [31] – e.g., we do not include primary prevention (vaccination) or detail the specifics of HPV infection and clearance – it was constructed to maximize the locally-available information and to gain locally-relevant policy and program insights. Markov models like this one are fundamentally decision trees, showing the ultimate outcomes that result from a series of probabilities. Owing to their simplicity – which we argue is a necessity due to the lack of robust data to parameterize a more complex model and an advantage for drawing relatively easy-to-explain findings that may resonate with diverse audiences that include non-technical experts – they cannot trace specific causes of outcomes and may lack some of the heterogeneity and variation in trajectories that other models offer.

We also note that models such as this one, and the scenarios that they analyze, may not fully capture the role of health system and implementation constraints or costs. This model represents per-woman increases in screening and treatment as a linear function, which smooths out the necessary capital investments to build more health facilities or hire additional health workers (each of which would be incurred in a nonlinear “step function”). It also does not consider the costs of achieving these increases in screening and treatment coverage: for example, if behavioral interventions or implementation strategies are needed in order to decrease loss to follow-up after a positive screening result, these may incur programmatic cost beyond the clinical ingredients reflected in this model. We strongly recommend that future analyses take a more nuanced approach to modeling health system constraints and costs and consider the costs of achieving the targets represented by the modeled scenarios. In addition, future models should strive toward disaggregated budget impact analyses so policymakers and other stakeholders can better understand the additional costs needed specifically for different prevention activities.

Some additional analytic limitations should be noted. First, the model was parameterized using data from a number of different African locations so very local heterogeneities may not be well-captured. Second, because cancer treatment is relatively unavailable in Malawi, we assumed that very few women would be treated; therefore, our analyses did not “save” these costs when lesions were prevented from progressing to cancer due to expanded “screen and treat.” The health gains were captured, but the cost savings were more limited than what would have been seen in an analysis from a higher-income context with greater utilization of cancer care. Third, we focus on a cohort of 30-year old women but many countries recommend an earlier starting age for screening. Fourth, although stratification by HIV status – both CD4 count and whether on ART – is an innovative aspect of this analysis, there may be misclassification error if the underlying data are not valid; and the model over-simplifies women’s experience living with HIV by not shifting them between CD4 count levels over time. It is possible that recently-implemented and highly effective HIV programs (like widespread testing and test-and-treat approaches) are identifying HIV infections before women are symptomatic and therefore they begin with low CD4 counts and experience slow disease progression – but future analyses using more granular approaches like microsimulation models should allow for HIV disease progression as well. Lastly, if certain baseline estimates are incorrect then the results of this model may be inaccurate. The sensitivity analyses presented here show the potential impact of such estimation errors. For example, the model was parameterized using 2015 data from the UNFPA and MOH about coverage of “screen and treat” but other data sources suggest that this may be much lower – for example, a household survey conducted in 2015 found that only approximately 11% of women without HIV, and 22% of women living with HIV were ever screened for cervical cancer [28]. Similarly, if the estimates of thermocoagulation effectiveness lack accuracy, the modeled estimates would be incorrect. Models like this one should therefore be repeated as more up-to-date information about “screen and treat” is collected particularly in high-burden settings like Malawi.

## Conclusions

This analysis demonstrates the cost-effectiveness of thermocoagulation as a cervical cancer treatment approach and underscores the importance of investing in universal treatment: in settings where women are screened infrequently, it would be more impactful to invest in expanding treatment for women who screen positive rather than encouraging women to screen more frequently. Screening schedules that differ by a woman’s HIV status would also be a prudent use of resources. This model offers new insights but further work is needed, including expanding such models to account for the implementation of HPV DNA testing as a primary screening approach, following recently-updated World Health Organization recommendations [60].

## Data Availability

Not applicable; all data used for this analysis are from existing secondary sources.

## Abbreviations

ART: Antiretroviral therapy
CIN: Cervical intraepithelial neoplasia
HPV: Human papillomavirus
ICER: Incremental cost-effectiveness ratio
LMICs: Low- and middle-income countries
QALY: Quality-adjusted life year
VIA: Visual inspection with acetic acid
WHO: World Health Organization
